# Identification of the key ferroptosis-related genes involved in sepsis progression and experimental validation *in vivo*

**DOI:** 10.3389/fphar.2022.940261

**Published:** 2022-08-11

**Authors:** Zhixi Li, Yongjing Yu, Chang Liu, Guangmin Chen, Weidong Gong, Juan Luo, Ziyong Yue

**Affiliations:** ^1^ Heilongjiang Province Key Laboratory of Research on Anesthesiology and Critical Care Medicine, Harbin, China; ^2^ The Key Laboratory of Myocardial Ischemia Organization, Chinese Ministry of Education, Harbin, China; ^3^ Department of Anesthesiology, The Second Affiliated Hospital of Harbin Medical University, Harbin, China; ^4^ Department of Anesthesiology, The First Affiliated Hospital of Harbin Medical University, Harbin, China

**Keywords:** ferroptosis, sepsis, acute lung injury, bioinformatics, MAPK signaling pathway, acupoint catgut embedding

## Abstract

**Background:** Ferroptosis has a vital role in sepsis, but the mechanism is not known. Understanding the mechanism of ferroptosis during sepsis will aid in developing improved therapeutic strategies.

**Methods:** We used the Gene Expression Omnibus database and FerrDb database to obtain ferroptosis-related differentially expressed genes (DEGs) between sepsis patients and healthy volunteers (HVs). Analyses of PPI networks, functional enrichment, as well as use of the MCODE algorithm were used to identify key ferroptosis-related DEGs. Expression of key ferroptosis-related DEGs was verified using: GSE57065 and GSE65682 datasets; rats in which ferroptosis was induced with erastin; sepsis-induced acute lung injury (siALI) rats. The effects of acupoint catgut embedding (ACE) on ferroptosis and expression of key ferroptosis-related DEGs in the lungs of siALI rats were also observed. A Cox proportional hazard model was used to verify the effect of key ferroptosis-related DEGs on the survival of sepsis patients. Cytoscape was used to construct ceRNA networks and gene–transcription factor networks.

**Results:** Between sepsis patients and HVs, we identified 33 ferroptosis-related DEGs. According to analyses of PPI networks and the MCODE algorithm, we obtained four modules, of which the most significant module contained nine ferroptosis-related DEGs. Functional-enrichment analyses showed that four of the nine DEGs were enriched in the MAPK signaling pathway: *MAPK14*, *VEGFA*, *TGFBR1*, and *DUSP1*. We verified expression of these four genes in GSE57065 and GSE65682 datasets and ferroptosis rats. In addition, expression of these four genes and that of the oxidative-stress indicators GSSG and MDA was upregulated, and glutathione peroxidase-4 (GPX4) expression was downregulated, in siALI rats, but ACE reversed these changes. The Cox proportional hazard model showed that survival of sepsis patients in the high-risk group was shorter than that in the low-risk group. We found that the XIST−hsa-let-7b-5p−TGFBR1/DUSP1 ceRNA network and transcription factor E2F1 may be important regulators of these four DEGs.

**Conclusion:** Our results suggest that *MAPK14*, *VEGFA*, *TGFBR1*, and *DUSP1* may be key regulatory targets of ferroptosis in sepsis, and that ACE pretreatment may be antioxidant treatment for sepsis and alleviate ferroptosis. These findings provide a basis for further ferroptosis-related study in sepsis and provide new targets for its treatment.

## Introduction

Sepsis has a global mortality rate of 20%, affects 49 million patients, and causes 11 million deaths per year: it is a significant healthcare problem worldwide (Rudd et al., 2020). However, how sepsis occurs is very complex, and has not been elucidated fully (Tian et al., 2019). Programmed cell death plays an essential part in sepsis and organ dysfunction, and is an essential target for its intervention, including ferroptosis, pyroptosis, and apoptosis (Li N. et al., 2020). Among them, ferroptosis is a new form of programmed cell death and is one of the leading causes of organ dysfunction due to sepsis (Li J. et al., 2021), which has attracted a great deal of interest among researchers.

Ferroptosis is one of the main pathogenic mechanisms of ischemia–reperfusion injury, degenerative diseases, and carcinogenesis (Chen et al., 2020). It is characterized by iron-dependent lipid peroxidation and, in addition, by a decrease in the expression of the antioxidant systems glutathione and glutathione peroxidase 4 (GPX4) (Imai et al., 2017). The decrease in GPX4 activity leads to oxidative cell death. In recent years, ferroptosis has been found to play an important role in the multi-organ dysfunction caused by sepsis, and inhibition of ferroptosis can alleviate acute injury to the kidney (Mishima et al., 2020), lung (Li J. et al., 2021), and liver (Wei et al., 2020). However, the specific molecular mechanisms of ferroptosis in sepsis and the resulting organ dysfunction are unknown.

Bioinformatics analysis (BA) has been used to explore the pathogenesis of diseases (Goh, 2018), however, there are no bioinformatics-based studies on the mechanism of ferroptosis occurrence in sepsis. By analyzing the dataset from the GEO database and intersecting it with the FerrDB database, we identified ferroptosis-related differentially expressed genes (DEGs) in sepsis patients. Since the new definition of sepsis places more emphasis on organ dysfunction caused by infection, and the lung is often the most susceptible organ to be involved in the early stage of sepsis (Heming et al., 2021), we used cecum ligation puncture (CLP) to construct sepsis-induced acute lung injury (siALI) animal models to verify our results obtained by bioinformatics analysis. Acupuncture is one of the most important therapeutic tools in treating various inflammatory diseases in traditional Chinese medicine (Oh and Kim, 2021). Acupoint catgut embedding (ACE) is an extension of acupuncture with a more sustained therapeutic effect, and studies have shown ACE to inhibit inflammation-based pain by inhibiting the MAPK signaling pathway (Du et al., 2017). Based on the above theory, this study explored the effect of ACE on ferroptosis in sepsis. In addition, we constructed a Cox proportional hazard model using target genes to observe its effect on the survival of sepsis patients. Finally, a gene−microRNA−long non-coding RNA (gene−miRNA−lncRNA) network was constructed and transcription factors (TFs) were predicted based on key ferroptosis-related DEGs. Our results could aid understanding of ferroptosis in sepsis and broaden treatment options for sepsis.

## Materials and methods

### Microarray data

We visited the Gene Expression Omnibus (GEO) database (www.ncbi.nlm.nih.gov/geo/) and obtained the datasets GSE95233, GSE57065, and GSE65682. Fifty-one patients with sepsis and 22 healthy volunteers (HVs) were in GSE95233. GSE57065 comprised 28 patients with sepsis and 25 HVs. GSE65682 comprised 760 patients with sepsis and 42 HVs. GSE95233 was the analysis dataset; GSE57065 and GSE65682 were the validation datasets. In addition, GSE65682 was the clinical-data source for sepsis patients in the present study for survival analyses.

### Normalization of microarray data

After obtaining the data, the data were corrected and normalized using R software. The probes corresponding to multiple molecules were removed, and when probes corresponding to the same molecule were encountered, only the probe with the highest signal value was retained.

### Identification of ferroptosis-related differentially expressed genes

We used GEO2R (www.ncbi.nlm.nih.gov/geo/geo2r/; 3 January 2022) to compare the gene-expression data between the sepsis patients and HVs of GSE95233 with the screening criteria of DEGs of |log_2_ (fold-change)| >1 and *p*_adjusted_ < 0.05. Ultimately, the ferroptosis-related genes obtained from FerrDb (www.zhounan.org/ferrdb/index.html/) were intersected with the DEGs of GSE95233 to identify ferroptosis-related DEGs.

### Analyses of functional enrichment

The packages clusterProfiler (statistical analysis and visualization) and org. Hs.eg.db (ID conversion) were loaded using R in order to perform Genome-Ontology (GO) analysis and Kyoto Encyclopedia of Genes and Genomes (KEGG) analysis of ferroptosis-related DEGs (significant as *p* < 0.05).

### Protein–protein interaction networks

To obtain PPI networks for ferroptosis-related DEGs, we uploaded ferroptosis-related DEGs to the Search Tool for the Retrieval of Interacting Genes/Proteins (STRING) database (www.string-db.org/) for prediction of PPI networks (interaction score >0.4). We used Cytoscape v.3.7.2 and the MCODE plug-in to undertake clustering analyses of ferroptosis-related DEGs.

### Dataset validation of ferroptosis-related differentially expressed genes

First, we downloaded the GPL570 and GPL13667 gene-annotation files and used Perl scripts to annotate the raw data of GSE57065 and GSE65682 to obtain the gene-expression matrix of these two datasets. We analyzed expression of the key ferroptosis-related DEGs in GSE57065 and GSE65682 to verify the accuracy of differences in target-gene expression.

### Animals and grouping

The animal study was reviewed and approved by the Animal Care and Use Committee of the Second Affiliated Hospital of Harbin Medical University (Approval No. SYDW 2021-087, Nangang, China). Male adult Sprague–Dawley rats (8–9 weeks) were purchased from the Experimentation Center of the Second Affiliated Hospital of Harbin Medical University. Rats were assigned randomly to three groups: sham, cecum ligation puncture (CLP), and CLP + ACE.

### Surgical procedure for rats with sepsis-induced acute lung injury

We used CLP to construct a model of siALI to verify BA results. An siALI model in rats was constructed in accordance with previous studies (Rittirsch et al., 2009). In brief, isoflurane was used to anesthetize rats. Using a 5-0 surgical suture to ligate the cecum. An 18-G puncture needle was employed to penetrate the midpoint of the ligated cecum. We squeezed out an appropriate amount of intestinal contents to ensure the patency of the penetration site, and returned the cecum to the abdominal cavity. In the sham group, the cecum is not ligated and punctured and sent back to the abdominal cavity, and the remaining procedures were identical to those undertaken in the CLP group.

### Acupoint catgut embedding

In reference to the *Rat Acupoint Atlas*, we selected the bilateral Feishu acupoints (BL13: 3-mm adjacent to the third thoracic vertebra on the back) combined with the bilateral Zusanli acupoints (ST36; 5-mm adjacent to the anterior tibial tuberosity) as treatment acupoints. ACE was undertaken 24-h before CLP. Rats in the sham group and CLP group received the same level of anesthesia and puncture of the same site, but without catgut embedding.

### Erastin administration

To revalidate the correlation between key ferroptosis-related DEGs and ferroptosis, an animal model of ferroptosis was constructed using the classical ferroptosis inducer erastin with reference to previous studies (Zhao et al., 2021). This was done by randomly dividing the rats into two groups: Solvent group (*n* = 6) and erastin group (*n* = 6). The rats were injected intraperitoneally with 25 mg/kg of Erastin (MCE) or solvent (5% DMSO + 40% PEG400 + 5% Tween-80 + 50% saline) at 12-h intervals for 2 days.

### ELISA analysis

Blood was collected from rats *via* the abdominal aorta 24 h after CLP, and samples were stored at −20°C until use (*n* = 6 per group). Serum levels of TNF-α and IL-6 were determined using the respective ELISA kits (Jingkang, Beijing, China).

### Wet/dry weight ratios

The exudate from the superficial layer of the lower lobe of the right lung of rats were blotted out. We weighed them to obtain the wet weight (W), followed by baking in an oven at 70°C for 48 h. Then, they were weighed to obtain the dry weight (D). Next, the W**/**D ratio was calculated, which we used to evaluate the degree of pulmonary edema (*n* = 6 per group).

### Histology

The right middle lobe of the rat lung was isolated and immersed in paraformaldehyde for 24 h. Subsequently, we stained lung tissue using hematoxylin & eosin (H&E). Light microscopy was employed to observe histopathological changes in the lungs by two researchers blinded to the study protocol. Lung tissue damage was scored with reference to the scoring method of the previous study (Matute-Bello et al., 2011). (A) neutrophil aggregation in alveolar lumina; (B) neutrophil infiltration in the interstitial lung; (C) formation of hyaline membranes; (D) formation of protein fragments in alveolar lumina; (E) thickening of the alveolar wall. The final score of the pathological section was calculated according to the formula [(20 × A) + (14 × B) + (7 × C)+ (7 × D) + (2 × E)/(number of fields × 100)]. Each group contained ≥20 random high-power fields (×400 magnification).

### Western blotting

Rats were executed 24 h after CLP. The right lobe of the lung was taken for testing (*n* = 6 per group). Protein was extracted using a mixture of RIPA lysis buffer. Primary antibodies against GPX4 (catalog number: ab125066; Abcam, Cambridge, UK), β-actin (AC026; ABclonal, Wuhan, China) and anti-rabbit secondary antibody (Ab6721; Abcam) were used.

### Malondialdehyde detection

A lipid peroxidation malondialdehyde assay kit (Beyotime, Shanghai, China) was used to measure malondialdehyde content in lungs following manufacturer instructions. Absorbance measurements were taken at 532 nm (*n* = 5 per group).

### Detection of glutathione disulfide

Oxidized GSSG is a common marker for measuring levels of oxidative stress. The GSSG level in lungs was quantified using a detection kit (S0053; Beyotime Institute of Biotechnology). Absorbance measurements were taken at 412 nm (*n* = 5 per group).

### qRT-PCR

TRIzol™ Reagent (Invitrogen, United States) was used to extract RNA from the lung tissues (*n* = 5 per group). Complementary-DNA was synthesized from 1 μg of total RNA (Toyobo, Osaka, Japan) on an S1000 system (Bio-Rad Laboratories). Quantitative RT-PCR was performed using AceQ Universal SYBR qPCR Master Mix (Q511-02/03, Vazyme, Nanjing, China). The messenger (m) RNA level was standardized to that of endogenous glyceraldehyde 3-phosphate dehydrogenase. Expression of target genes was analyzed by the 2−^△△ct^ method. The primer sequences for rat samples were:

*Mapk14*-F:5′- GAT​GCC​AAG​CCA​TGA​GGC​AA.
*Mapk14*-R:5′- GGG​TCG​TGG​TAC​TGA​GCA​AA.
*Vegfa*-F:5′- ACT​CAT​CAG​CCA​GGG​AGT​CT.
*Vegfa*-R:5′- GGG​AGT​GAA​GGA​GCA​ACC​TC.
*Tgfbr1*-F:5′- ACT​CCC​AAC​TAC​AGA​AAA​GCA.
*Tgfbr1*-R:5′- AAG​GGC​GAT​CTA​GTG​AGG​GA.
*Dusp1*-F:5′- ATA​TCG​TGC​CGA​ACA​CCG​AA.
*Dusp1*-R:5′- ACG​CTT​CAT​ATC​CTC​CTT​GG.
*Gapdh*-F:5′- GTC​GGT​GTG​AAC​GGA​TTT.
*Gapdh*-R:5′- ACT​CCA​CGA​CGT​ACT​CAG​C.

### Construction and validation of cox proportional hazard model

Considering the importance of the key ferroptosis-related DEGs, the Cox proportional hazard model was constructed. The GSE65682 dataset was first screened to remove samples with missing prognostic data, and combined with normalized gene expression data, 479 samples were finally obtained for subsequent analysis. The 479 samples were randomly grouped in a 1:1 ratio into the training group (*n* = 240) and the test group (*n* = 239). The “survival” package and “survminer” package were loaded in the R software, and the Cox proportional hazard model was constructed in the training group to score the risk of sepsis patients.

### Survival analyses

Patients were classified into high-risk and low-risk groups based on the median risk score. Kaplan-Meier analysis was used to explore the prognostic significance of the ferroptosis-related gene signature on sepsis patients. The log-rank test was employed to analyze the difference in survival probability between the two groups.

### Construction of the ceRNA network and the target gene-transcription factor network

We utilized miRDB, miRmap, miRWalk, miRTarBase, RNA22, and TargetScan databases to predict miRNAs that could regulate key ferroptosis-related DEGs. Prediction of a miRNA for each target referred to the prediction results of ≥4 databases. After obtaining the predicted miRNAs, prediction of lncRNAs was done on the selected miRNAs using starBase (https://starbase.sysu.edu.cn/; mammalian, human h19 genome, strict stringency (≥5) of CLIP-Data and with/without degradome data). We used NetworkAnalyst (www.networkanalyst.ca/), referring to the JASPAR database, to predict TFs that could regulate key ferroptosis-related DEGs.

### Statistical analyses

Statistical analyses were done using SPSS 25.0 (IBM, Armonk, NY, United States), Prism 6.01 (GraphPad, San Diego, CA, United States), and R. Data were displayed as mean ± SD. The Student’s *t*-test was used for comparison between two groups. One-way ANOVA was employed for comparison between multiple groups, and the *post hoc* multiple comparisons least significant difference and Student–Newman–Keuls model were selected for analyses, *p* < 0.05 was considered significant.

## Results

### Ferroptosis-related differentially expressed genes

**FIGURE 1:**
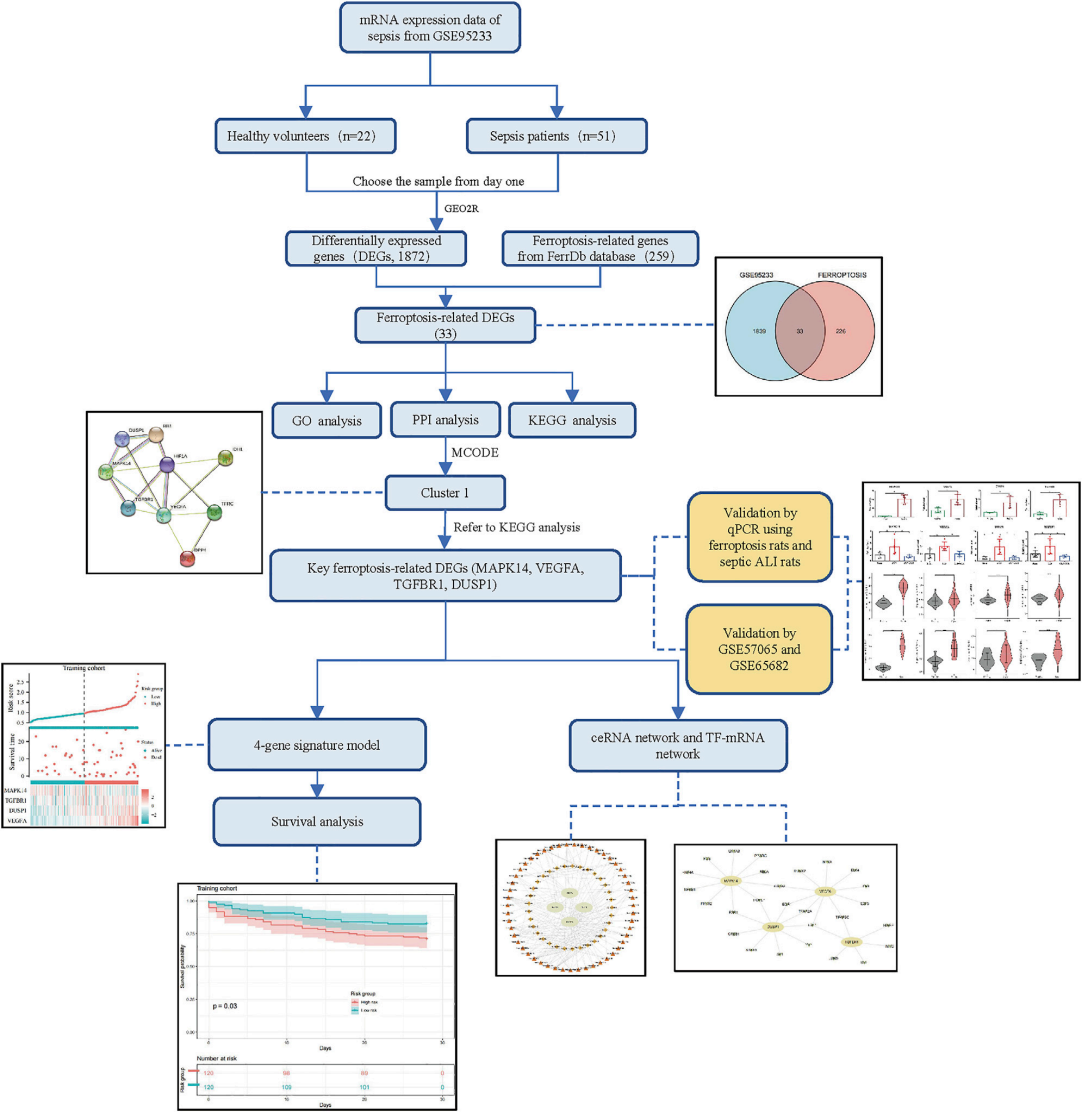
Flowchart of our study.

**FIGURE 2:**
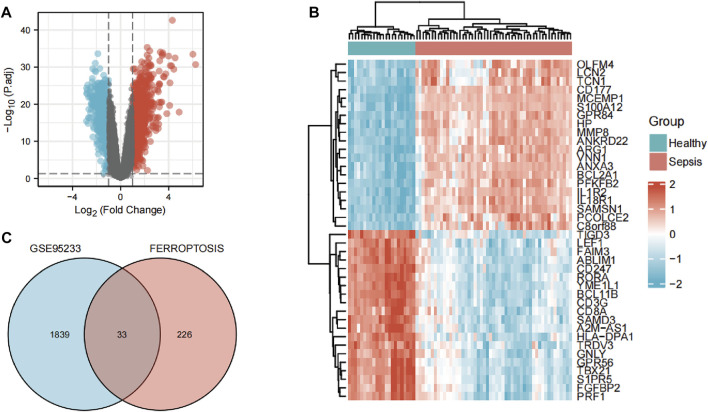
Identification of ferroptosis-related differentially expressed genes (DEGs). **(A)** Volcano plots of DEGs. **(B)** Heatmap of DEGs identified in GSE95233. **(C)** Venn diagram of ferroptosis-related DEGs.

The flowchart of our study is displayed as Figure 1. By analyzing GSE95233 using GEO2R, we obtained 1872 DEGs: expression of 1070 genes was upregulated and expression of 802 genes was downregulated (Figures 2A,B). We identified 259 ferroptosis-related genes from FerrDb, which intersected with 1872 DEGs and, finally, we obtained 33 ferroptosis-related DEGs (Figure 2C): 27 showed upregulated expression and 6 showed downregulated expression (Table 1).

**TABLE 1:** Ferroptosis-related differentially expressed genes of sepsis.

Gene symbol	Adi.P.Val	logFC	Gene title	ID
SLC38A1	7.21E-21	−1.91	solute carrier family 38 member 1	218237_s_at
DPP4	8.39E-21	−1.85	dipeptidyl peptidase 4	203716_s_at
TP53	1.05E-22	−1.68	tumor protein p53	201746_at
PEBP1	6.67E-18	−1.55	phosphatidylethanolamine binding protein 1	210825_s_at
LPIN1	2.51E-18	−1.45	lipin 1	212274_at
ATM	7.54E-16	−1.18	ATM serine/threonine kinase	208442_s_at
DUSP1	2.46E-15	1.35	dual specificity phosphatase 1	219963_at
RB1	4.94E-11	1.05	RB transcriptional corepressor 1	203132_at
IDH1	1.29E-15	1.17	isocitrate dehydrogenase (NADP (+)) 1, cytosolic	1555037_a_at
VEGFA	5.98E-08	1.08	vascular endothelial growth factor A	210512_s_at
MAFG	1.82E-17	1.08	MAF bZIP transcription factor G	224466_s_at
TGFBR1	1.51E-08	1.08	transforming growth factor beta receptor 1	224793_s_at
TLR4	4.64E-13	1.08	toll like receptor 4	224341_x_at
ACSL3	1.91E-13	1.27	acyl-CoA synthetase long-chain family member 3	201660_at
CD44	6.75E-10	1.13	CD44 molecule (Indian blood group)	234418_x_at
CAPG	1.32E-13	1.15	capping actin protein, gelsolin like	201850_at
CISD2	1.91E-09	1.15	CDGSH iron sulfur domain 2	226686_at
TFRC	1.31E-06	1.18	transferrin receptor	208691_at
ACVR1B	2.80E-21	1.51	activin A receptor type 1B	213198_at
HIF1A	1.61E-12	1.21	hypoxia inducible factor 1 alpha subunit	200989_at
AURKA	7.12E-08	1.24	aurora kinase A	208079_s_at
ALOX5	3.63E-21	1.55	arachidonate 5-lipoxygenase	213952_s_at
WIPI1	1.84E-21	1.72	WD repeat domain, phosphoinositide interacting 1	213836_s_at
MAPK14	1.35E-26	2.05	mitogen-activated protein kinase 14	211561_x_at
EMC2	3.34E-15	1.33	ER membrane protein complex subunit 2	203584_at
SLC2A3	9.15E-22	1.63	solute carrier family 2 member 3	202499_s_at
RRM2	3.41E-06	1.50	ribonucleotide reductase regulatory subunit M2	209773_s_at
SAT1	1.69E-19	1.40	spermidine/spermine N1-acetyltransferase 1	213988_s_at
SLC40A1	2.59E-16	1.55	solute carrier family 40 member 1	223044_at
EPAS1	1.08E-13	1.62	endothelial PAS domain protein 1	200878_at
CHMP5	2.14E-15	1.71	charged multivesicular body protein 5	218085_at
PGD	7.84E-27	1.83	phosphogluconate dehydrogenase	201118_at
ACSL4	1.60E-15	1.83	acyl-CoA synthetase long-chain family member 4	202422_s_at

### Enrichment analyses

**FIGURE 3:**
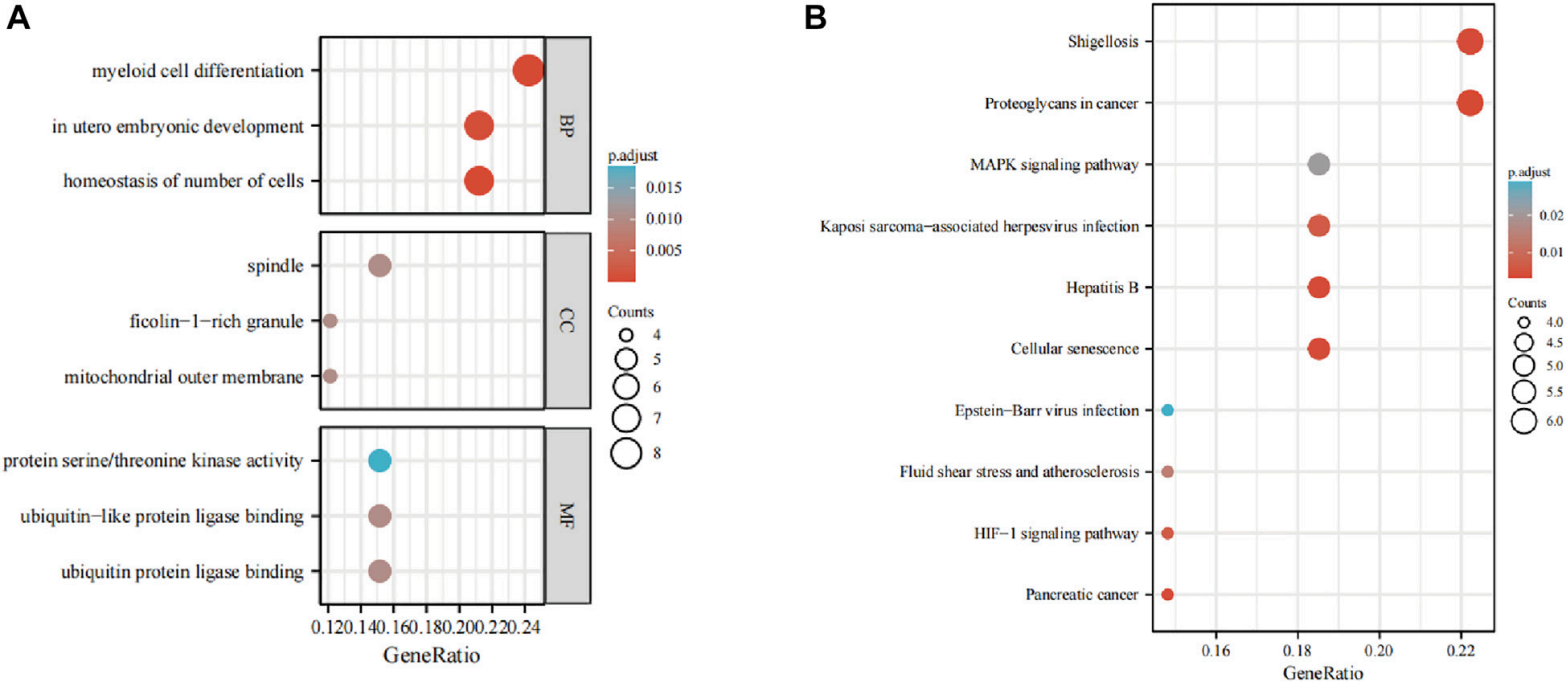
Enrichment analyses of **(A)** function of differentially expressed genes (DEGs) using the Gene Ontology database and **(B)** signaling pathways of DEGs using the Kyoto Encyclopedia of Genes and Genomes database.

The GO analysis showed the most significant enrichment terms to be “myeloid cell differentiation”, spindle and “protein serine/threonine kinase activity” individually for biological processes, cell component, and molecular function (Figure 3A). The KEGG analysis showed that “MAPK signaling pathway” was one of the major enrichment pathways for ferroptosis-related DEGs (Figure 3B).

### Analyses of PPI networks for ferroptosis-related differentially expressed genes

**FIGURE 4:**
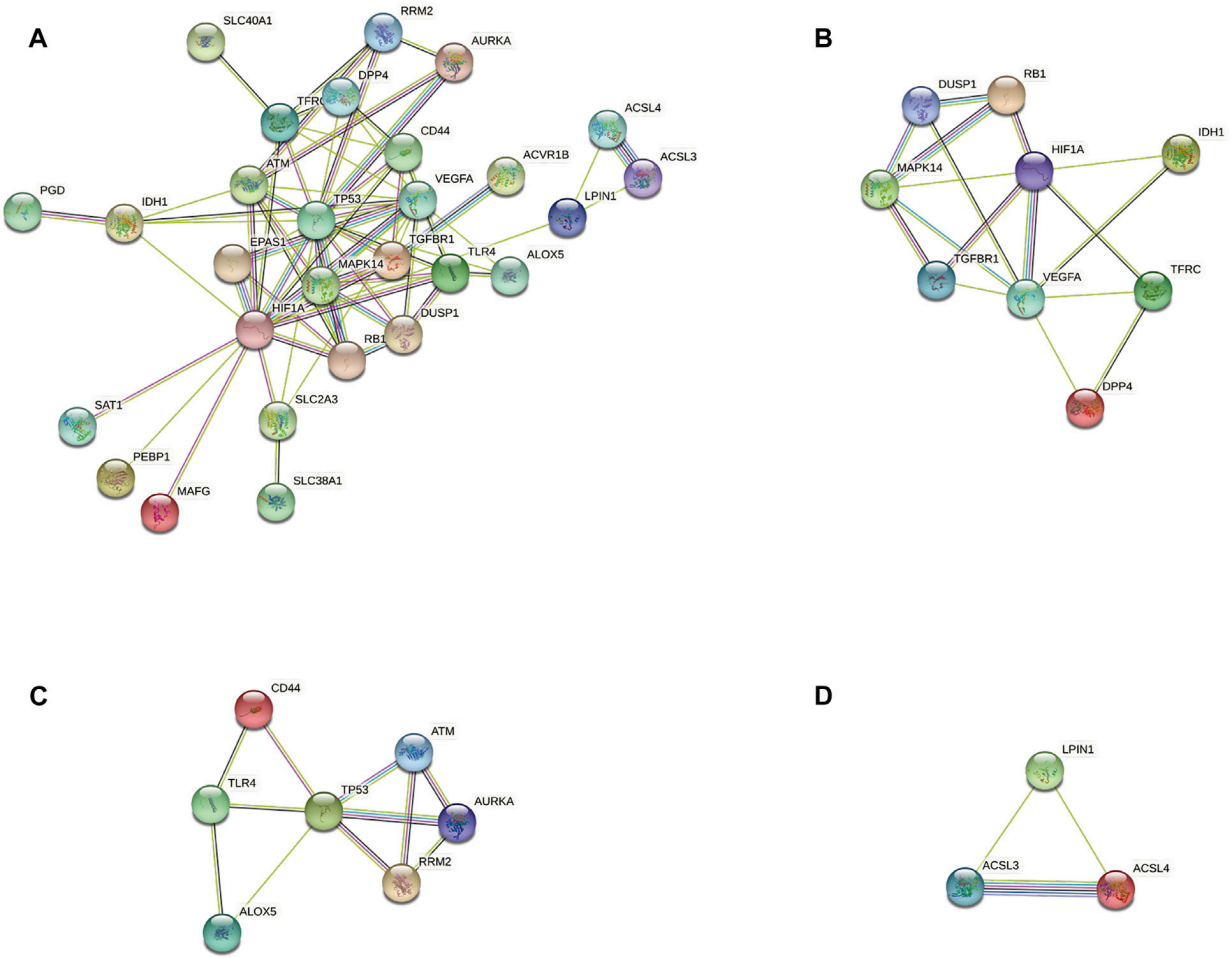
Protein–protein interaction network analyses of ferroptosis-related differentially expressed genes (DEGs). **(A)** PPI network of ferroptosis-related DEGs. **(B)** Cluster 1. **(C)** Cluster 2. **(D)** Cluster 3.

**FIGURE 5:**
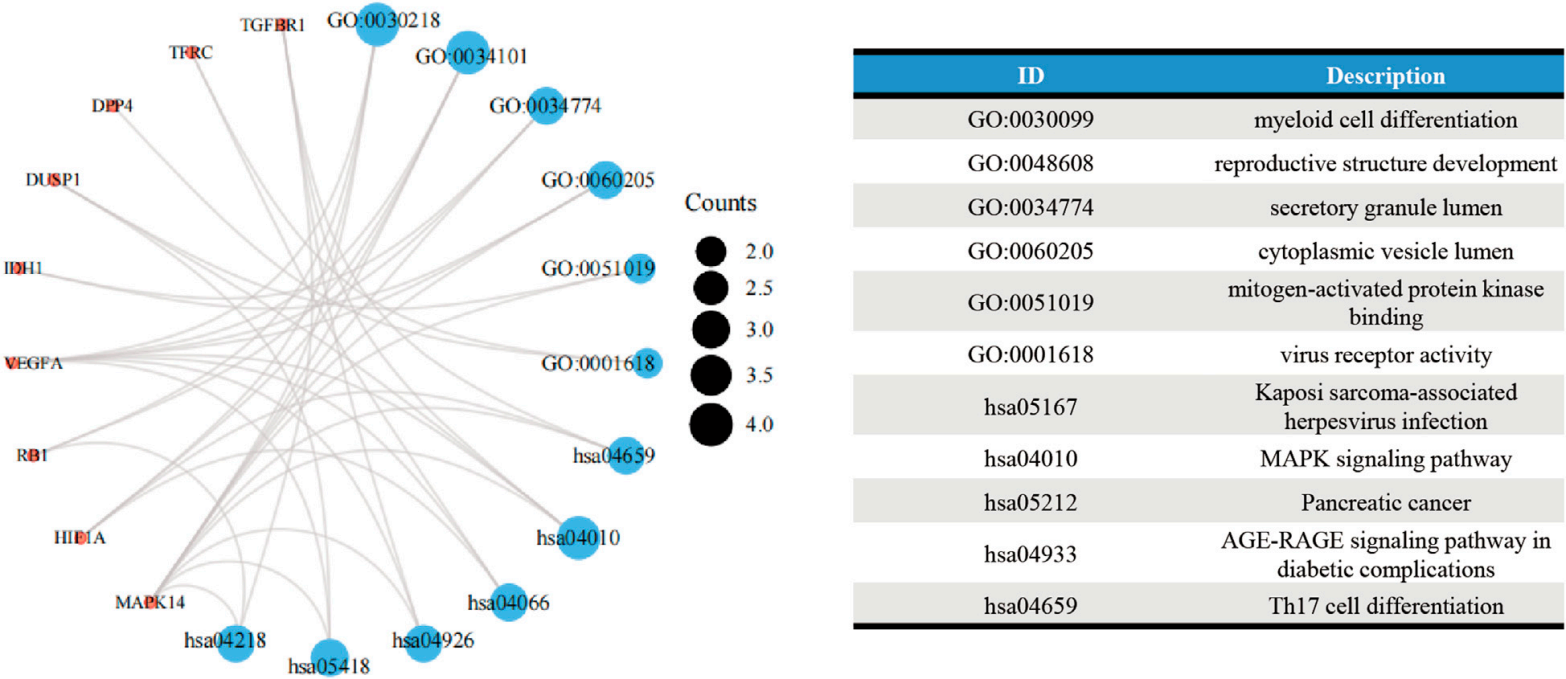
Functional-enrichment analyses of Cluster 1 genes.

According to the STRING database and Cytoscape, we obtained a PPI network comprising 33 nodes and 71 edges (Figure 4A). We used MCODE to identify key modules. We obtained four modules in this PPI network, in which the highest-scoring gene clusters were eight genes with upregulated expression (*MAPK14*, *TGFBR1*, *VEGFA*, *DUSP1*, *RB1*, *HIF1A*, *TFRC*, *IDH1*) and one gene with downregulated expression (*DPP4*) (Figure 4B). Based on functional-enrichment analysis of the cluster1 gene, we found four genes to be enriched in the MAPK signaling pathway. Considering the importance of this pathway for sepsis and ferroptosis, we chose these key ferroptosis-related DEGs for subsequent analyses: *MAPK14*, *VEGFA*, *TGFBR1*, and *DUSP1* (Figure 5).

### Verification of the key ferroptosis-related differentially expressed genes

**FIGURE 6:**
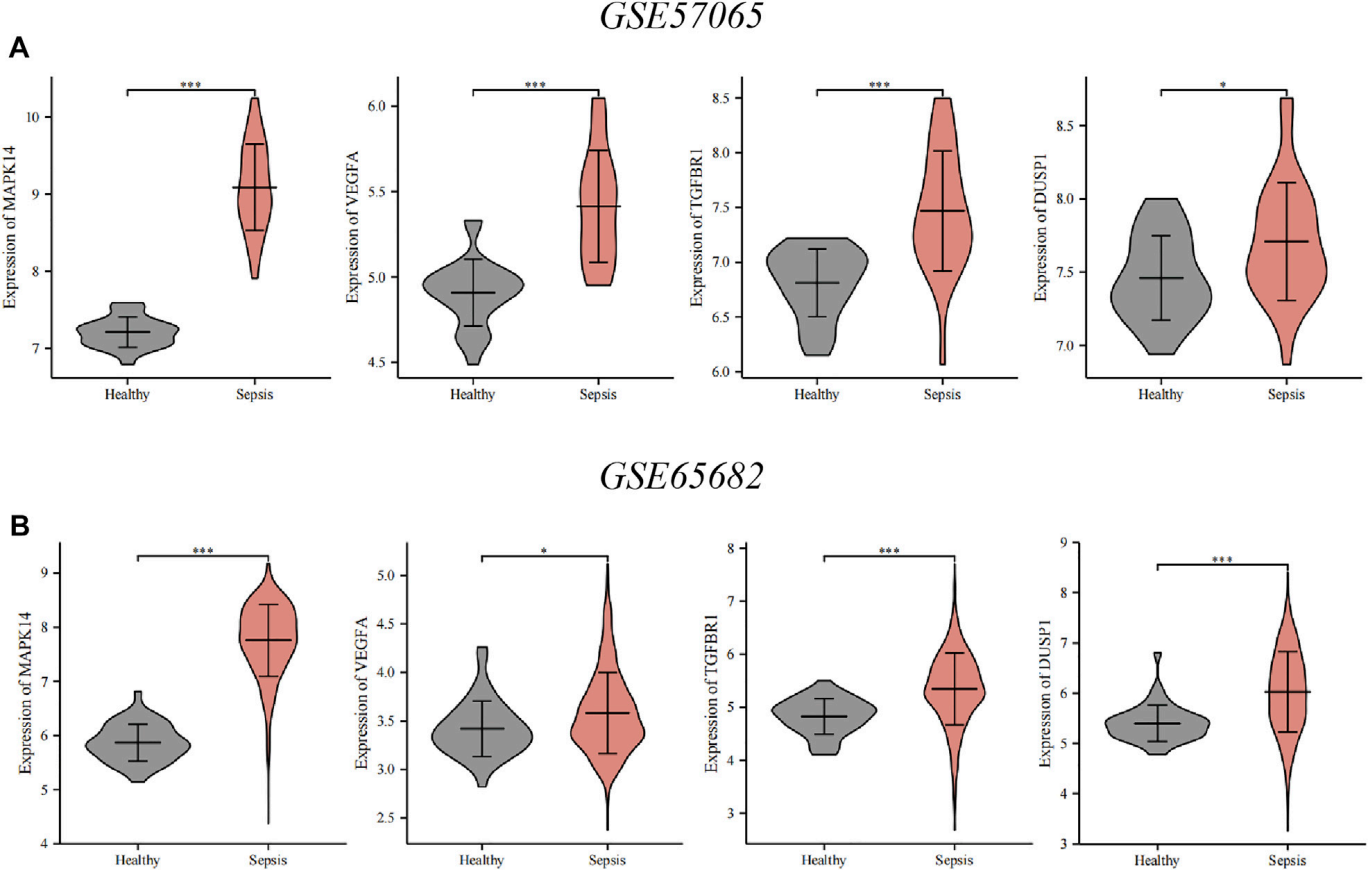
Verification of the key ferroptosis-related differentially expressed genes (DEGs) using datasets from the GEO database. **(A)** Verification by the GSE57065 dataset. Compared with healthy volunteers, expression of all key ferroptosis-related DEGs was upregulated in sepsis patients. **(B)** Verification by the GSE65682 dataset. Compared with healthy volunteers, expression of all key ferroptosis-related DEGs was upregulated in sepsis patients. ∗∗∗*p* < 0.001, ∗∗*p* < 0.01, ∗*p* < 0.05, ns, no significant difference.

To verify key ferroptosis-related DEGs, we selected GSE57065 and GSE65682 as validation datasets. mRNA expression of the four key ferroptosis-related DEGs was significantly higher in sepsis patients than that in HVs in GSE57065 and GSE65682 (Figure 6). Meanwhile, qRT-PCR results in erastin-induced ferroptosis animal models showed that these four key ferroptosis-related DEGs were highly expressed in the Erastin group compared to the Solvent group (Supplementary Figure S1).

### Effect of acupoint catgut embedding on sepsis-induced acute lung injury rats

**FIGURE 7:**
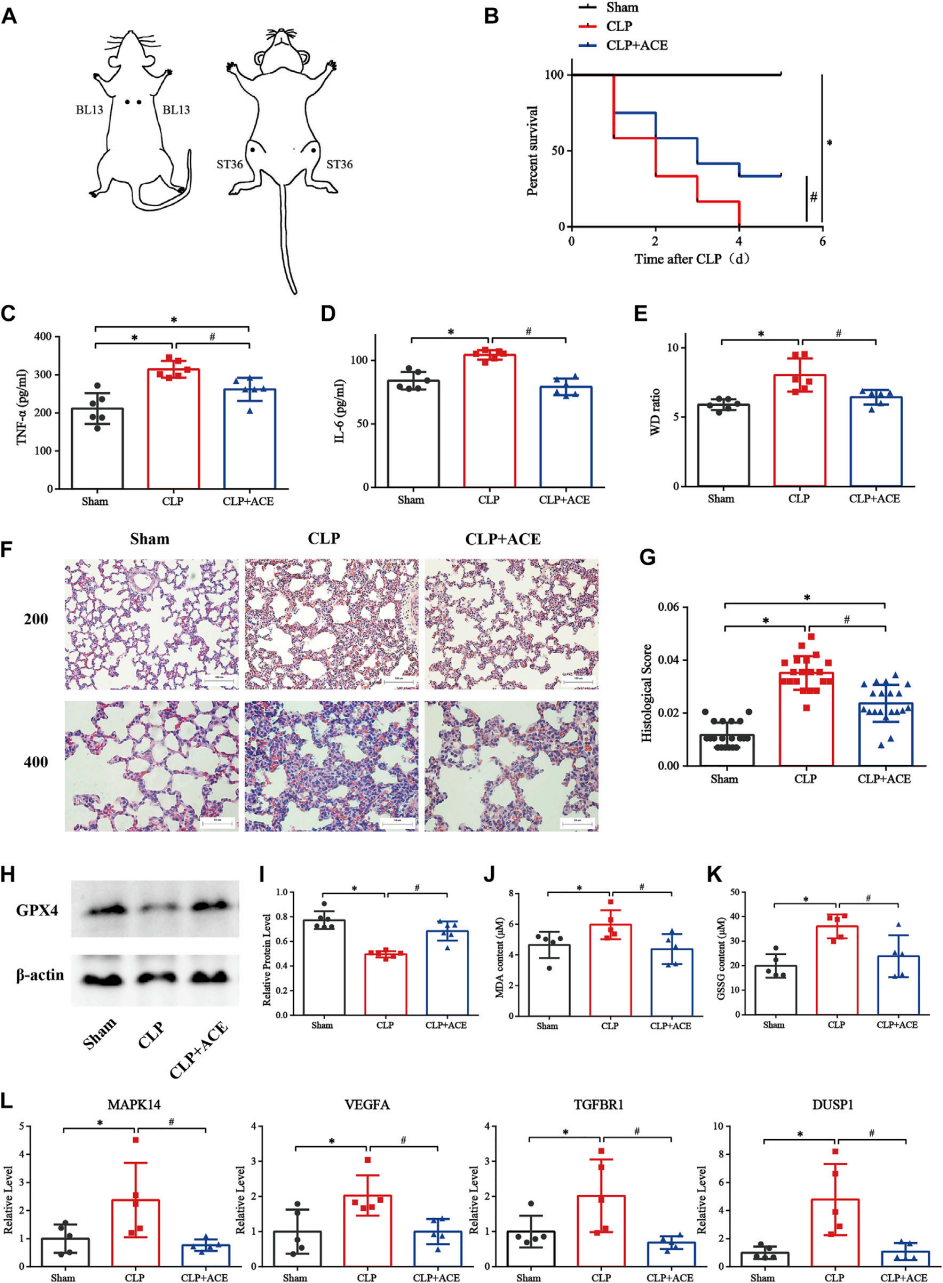
Effect of acupoint catgut embedding (ACE) on sepsis-induced acute lung injury (siALI) rats and ferroptosis. **(A)** Acupoint location (schematic). **(B)** ACE increased the 5-day survival of siALI rats (*n* = 12 per group). **(C)** TNF-α level in serum. **(D)** IL-6 level in serum. **(E)** W/D ratio in lungs. **(F)** H&E staining of pulmonary sections: scale bar is 50 µm or 100 µm. **(G)** Lung-injury scores. **(H,I)** Western blotting shows that ACE upregulated of GPX4 expression in the lungs of siALI rats. **(J,K)** Levels of GSSG and MDA were measured using assay kits. **(L)** qRT-PCR shows that expression of *MAPK14*, *VEGFA*, *TGFBR1*, and *DUSP1* was higher in siALI rats than that in the sham group. After ACE, expression of these four genes was downregulated. Data are the mean ± SD (*n* = 5 or 6 per group). ∗*p* < 0.05 *vs*. sham group, #*p* < 0.05 *vs*. CLP group.

As illustrated in Figures 7A,B, ACE enhanced survival within 5 days in siALI rats. The TNF-α and IL-6 were decreased under ACE treatment (Figures 7C,D). We calculated the W/D ratio and observed the pathological changes in lungs. The W/D ratio in the CLP group was significantly increased, whereas that in the ACE group was decreased (Figure 7E). The lung tissues of rats in the sham group had a normal structure. The lung tissues of rats in the CLP group were severely damaged with many inflammatory cells infiltrating the alveolar septum (and widening it), and alveolar and septal hemorrhage and higher-lung injury scores were observed. The above-mentioned manifestations were reduced significantly in ACE-group rats (Figures 7F,G). These results suggested that ACE could improve the severity of lung injury in siALI rats.

### Effect of acupoint catgut embedding on ferroptosis

The expression of GPX4 in the lung tissues of siALI rats was reduced significantly, whereas it was increased in the ACE group compared with CLP group. (Figures 7H,I). The levels of MDA and GSSG were increased in siALI rats and decreased in the ACE group (Figures 7J,K). In addition, the mRNA expression of *MAPK14*, *VEGFA*, *TGFBR1*, and *DUSP1* was significantly increased in the lungs of rats in the CLP group, while the expression of these genes was downregulated in the ACE group (Figure 7L). These results were consistent with BA trends. Taken together, these results suggested that the administration of ACE could alleviate ferroptosis in siALI rats, and validated the accuracy and importance of BA results.

### Construction of prognostic signature and survival analysis

**FIGURE 8:**
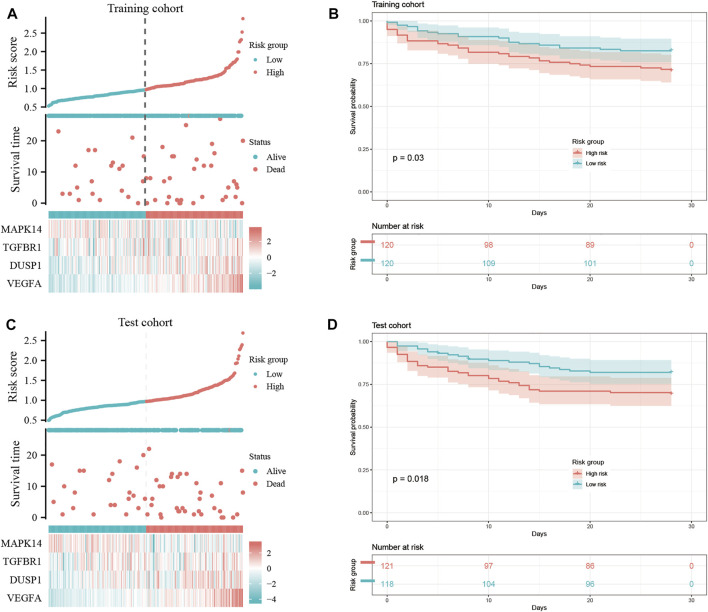
Construction of a “prognostic signature” and survival analyses. Risk scores **(A,C)** and survival outcome **(B,D)** of each case are shown.

We wished to ascertain if the four key ferroptosis-related DEGs could influence the prognosis of sepsis patients. We constructed a Cox proportional hazard model for risk scoring using these four key ferroptosis-related DEGs with data from training group. Then, we divided patients into high-risk and low-risk groups (Figures 8A,C) and observed the survival rate both in training group and test group. The survival rate of sepsis patients in the high-risk group was reduced within 28 days compared with that of sepsis patients in the low-risk group (Figures 8B,D). This result suggested that these four key ferroptosis-related DEGs may influence the prognosis of sepsis patients.

### Construction of a ceRNA network and transcription factor-gene network

**FIGURE 9:**
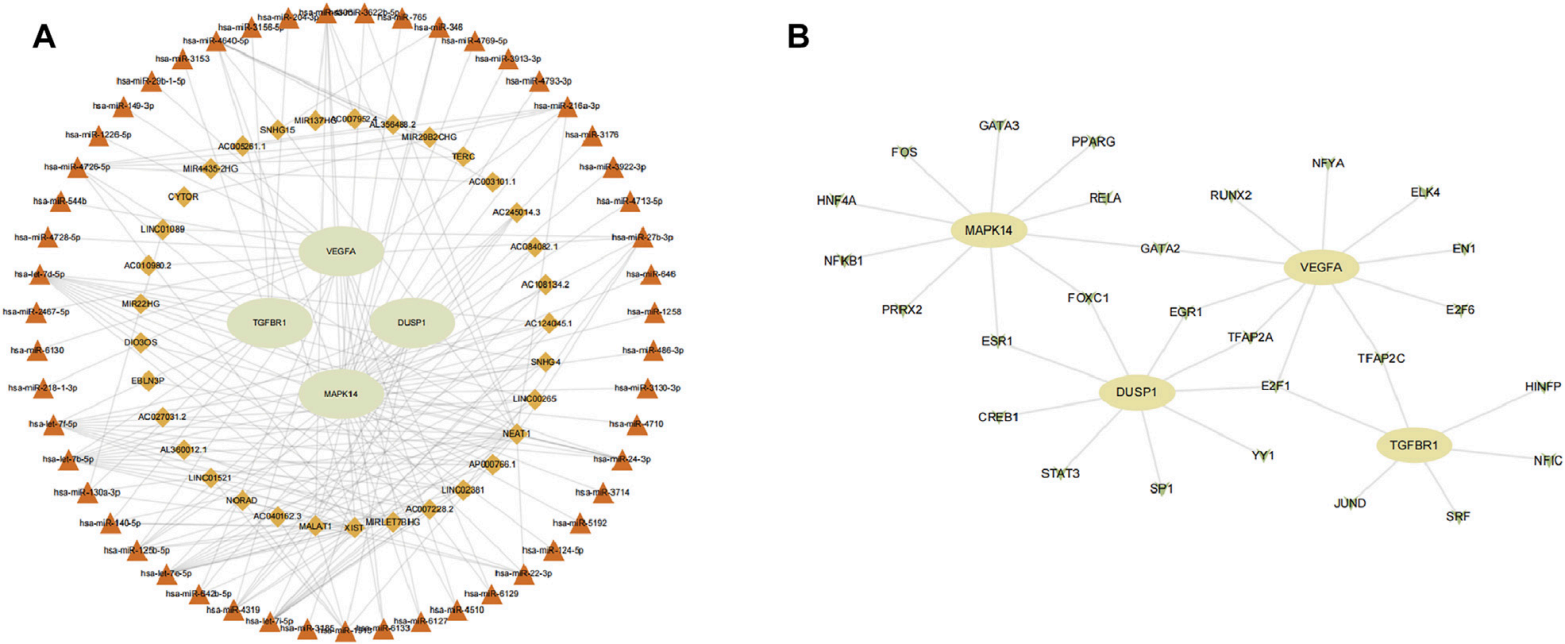
Network construction. **(A)** ceRNA network of key ferroptosis-related differentially expressed genes (DEGs). **(B)** TF–gene network of key ferroptosis-related DEGs. Ellipse nodes are genes, diamond nodes are lncRNAs, triangle nodes are miRNAs, and V nodes are TFs.

We aimed to predict the ceRNA networks that may be involved in regulating key ferroptosis-related DEGs. The results showed that we identified 51 target miRNAs and 114 mRNA−miRNAs. Six of the miRNAs could regulate more than one target gene (Table 2). Finally, we got the 34 target lncRNAs based on these miRNAs (Figure 9A). Among these six miRNAs, *hsa-let-7b-5p* could match to the most lncRNAs (*AC124045.1*, *SNHG4, LINC00265*, *AP000766.1*, *LINC02381*, *AC007228.2*, *MIRLET7BHG*, *XIST*). We also used NetworkAnalyst to predict the TFs that could regulate key ferroptosis-related DEGs: 27 target TFs that may be involved in regulating key ferroptosis-related DEGs were identified. Among these TFs, E2F transcription factor 1 (*E2F1*) could match the most target genes (Figure 9B).

**TABLE 2:** miRNAs and its target genes.

miRNA	Genes targeted by miRNA	Gene count
hsa-let-7b-5p	DUSP1, TGFBR1	2
hsa-miR-216a-3p	MAPK14, TGFBR1	2
hsa-miR-4510	MAPK14, VEGFA	2
hsa-miR-6127	MAPK14, VEGFA	2
hsa-miR-6129	MAPK14, VEGFA	2
hsa-miR-6133	MAPK14, VEGFA	2

## Discussion

Recent researches have revealed that ferroptosis plays an essential part in organ dysfunction due to sepsis. Inhibition of ferroptosis is considered an effective treatment for sepsis (Li N. et al., 2020). However, the specific molecular mechanisms underlying ferroptosis during sepsis are unknown.

In the current study, we identified key genes involved in the regulation of ferroptosis in sepsis. We identified 33 ferroptosis-related DEGs: 27 had upregulated expression and 6 had downregulated expression. The MAPK signaling pathway was one of the major pathways to be enriched. We chose siALI rats for experimental validation. Based on BA results, we selected ACE as a treatment method to observe its effect on ferroptosis and key ferroptosis-related DEGs. The results showed that ACE could alleviate ferroptosis in siALI rats and reverse the upregulation of key ferroptosis-related DEGs. In addition, we constructed a Cox proportional hazard model using the key ferroptosis-related DEGs to score the risk of sepsis patients: sepsis patients in the high-risk group had higher mortality within 28 days. Among the key ferroptosis-related DEGs we identified, several genes have not been mentioned before in the context of sepsis or ferroptosis. Our data provide a reference for study of the pathogenesis of sepsis and the resulting organ dysfunction.

Iron is important in various biochemical processes. If cells are stressed, iron accumulates within them (“iron overload”). This process leads to redox imbalance, production of lipid ROS, one of the characteristics of ferroptosis (Zhou et al., 2018) and, ultimately, to cell death. Activation of MAPK signaling pathway has been shown to lead to increased levels of oxidative stress, which triggers ferroptosis (Lv et al., 2020). Lipid ROS can also activate the MAPK signaling pathway (Nakamura et al., 2019). BA showed that the MAPK signaling pathway was enriched in ferroptosis-related DEGs, and most genes enriched in this pathway had upregulated expression, which is consistent with other researches (Nakamura et al., 2019). Hence, we hypothesized that the MAPK signaling pathway was activated by ferroptosis in sepsis, which aggravated the level of oxidative stress in the organism and increased ROS production, thus making the organism enter a “vicious cycle”.

Based on the above theory, we suggest that the MAPK pathway-related genes *MAPK14*, *VEGFA*, *TGFBR1* and *DUSP1* may be important regulatory targets of ferroptosis in sepsis. Mitogen-Activated Protein Kinase 14 (*MAPK14*) is a serine-threonine protein kinase that belongs to the p38-MAPK subfamily (Luo et al., 2016). It has been shown that *MAPK14* plays an important regulatory role in diseases (e.g., pneumonia, acute lung injury, diabetes mellitus) and that inhibition of this pathway is an effective measure to alleviate the level of the inflammatory response and oxidative stress in disease states (Jackson et al., 1998). In addition, the MAPK signaling pathway has been found to be involved in the regulation of ferroptosis in a variety of diseases, including tumors, cold stress, and osteoporosis (Lin et al., 2022; Song et al., 2022). Vascular Endothelial Growth Factor A (*VEGFA*) shows abundant expression in lungs (Leung et al., 1989; Mura et al., 2006). *VEGFA* may also be involved in the inflammatory response by inducing expression of intracellular adhesion molecules in endothelial cells, including E-selectin, intercellular adhesion molecule 1, and vascular cell adhesion molecule 1, which promote leukocyte adhesion (Kim et al., 2001). Studies have shown that anti-VEGF antibody alleviates the inflammatory response in mice suffering from sepsis *in vivo* and reduces vascular leak in lungs (Jeong et al., 2013). Transforming Growth Factor Beta Receptor 1 (*TGFBR1*) is a key component in transmission of extracellular stimuli to the downstream TGF-β signaling pathway, and has vital roles in the growth and development of the organism and homeostasis maintenance (Vander Ark et al., 2018). It was found that when a specific inhibitor of *TGFBR1* was used, the inflammatory response caused by LPS-stimulated macrophages was reduced (Chen et al., 2008). However, the current studies on *TGFBR1* in animal models of sepsis are scarce and deserve further exploration. Dual Specificity Phosphatase 1 (*DUSP1*) can regulate cell proliferation and division, and is also thought to be a central mediator of the inflammatory response (Hoppstädter and Ammit, 2019). When *DUSP1* is knocked out in septic mice, it leads to excessive release of inflammatory factors *in vivo* and a further increase in mortality (Hammer et al., 2010). Those results suggest that *DUSP1* may have a positive regulatory effect on the inflammatory response: this is contradictory to our results showing ACE to downregulate mRNA expression of *DUSP1*. This contradiction may be related to the post-transcriptional modification of *DUSP1* or post-translational modification at the protein level. No studies have shown that *MAPK14*, *VEGFA*, *TGFBR1* and *DUSP1* regulate ferroptosis in sepsis. Therefore, we hypothesize that these genes might influence ferroptosis in sepsis and the resulting organ dysfunction.

Lungs are the most susceptible organs to pathological changes during sepsis (Shankar-Hari et al., 2016). It is well established that patients with siALI have a higher mortality rate (Kumar, 2020). We employed siALI rats to validate the BA results and found that the expression of *MAPK14*, *VEGFA*, *TGFBR1*, and *DUSP1* was upregulated in the lungs of siALI rats. These four genes are enriched mainly in the MAPK signaling pathway, and acupoint stimulation can exert anti-inflammatory effects by inhibiting the MAPK signaling pathway (Zhang et al., 2021). Hence, we chose ACE to observe the effects of ACE on siALI rats. ACE has been shown to contribute to the control of inflammation in organisms and to promote recovery of organ function in disease states (Li D. et al., 2020; Zhou et al., 2021). Recent studies have shown that electroacupuncture stimulation (another form of acupoint stimulation) of ST36 can alleviate lipopolysaccharide-induced ferroptosis in mice with sepsis-induced ARDS by modulating the alpha7 nicotinic acetylcholine receptor (Zhang et al., 2022). Consistently, our study showed that ACE alleviated ferroptosis in the lungs of siALI rats. Interestingly, ACE also decreased mRNA expression of *MAPK14*, *VEGFA*, *TGFBR1*, and *DUSP1*, which further confirmed the results of our BA and suggested that regulation of ferroptosis in siALI rats by ACE might be related to these four genes. In addition, we constructed a Cox proportional hazard model using *MAPK14*, *VEGFA*, *TGFBR1*, and *DUSP1*: they influenced survival within 28 days in sepsis patients, which emphasizes their importance in sepsis pathogenesis.

To explore the factors that can influence the expression of key ferroptosis-related DEGs, we used the bioinformatics website to predict the miRNAs, lncRNAs, and TFs that could regulate the expression of key ferroptosis-related DEGs. It was shown that in sepsis, *hsa-let-7b-5p* was able to regulate neutrophil function and reduce organismal inflammation, and when hsa-let-7b-5p agonist was used, it reduced neutrophil recruitment in sepsis mice and alleviated organ dysfunction (Chen et al., 2021). Among the upstream lncRNAs of *hsa-let-7b-5p*, only *XIS*T has been found to be involved in sepsis regulation. Studies have shown that silencing or downregulation of XIST expression protects against sepsis-induced multiorgan dysfunction (Wang et al., 2021; Wang and Cao, 2022). Findings from those studies are consistent with our prediction that *XIST*−*hsa-let-7b*-*5p*−*TGFBR1*/*DUSP1* may be a vital ceRNA regulatory network in sepsis. In addition, the TF that could match the most target genes was *E2F1*. Studies have shown that downregulation of *E2F1* activates the NFκB signaling pathway, which leads to sepsis progression (Li B. et al., 2021). However, the above prediction has not yet been investigated and deserves further exploration.

We identified four ferroptosis-related genes that may play an important part in ferroptosis development in sepsis, and validated them from other datasets as well as animal models. A therapeutic approach that alleviates ferroptosis development in sepsis was also identified, which complements studies on the mechanism of action of ferroptosis in sepsis. However, our study had three main limitations. First, we could not validate our experimental results in human tissues due to the difficulty of obtaining samples. Second, we focused on four important genes among 33 ferroptosis-related DEGs but neglected to explore other genes: more comprehensive and in-depth studies are needed in the future. Third, we validated only the key ferroptosis-related gene signature in an internal dataset due to a lack of other relatively well-developed prognostic data from sepsis patients. Validation in an external dataset is needed. The results of our study need further confirmation from laboratory tests and clinical data.

## Conclusion

We identified four ferroptosis-related genes (*MAPK14*, *VEGFA*, *TGFBR1*, *DUSP1*) that may influence the pathological progression of sepsis and the resulting organ dysfunction. ACE pretreatment alleviated pulmonary ferroptosis in siALI rats, and maybe novel adjuvant therapy for sepsis patients. We predicted that the *XIST*−*hsa-let-7b-5p*−*TGFBR1*/*DUSP1* ceRNA network and transcription factor E2F1 might be important regulators of sepsis. Our results may extend knowledge of sepsis and provide novel ideas for treating it.

## Data Availability

The original contributions presented in the study are included in the article/Supplementary Material, further inquiries can be directed to the corresponding author.
