# Supplementary material for: Identification of the key ferroptosis-related genes involved in sepsis progression and experimental validation in vivo
Source: Front Pharmacol. 2022 Aug 11;13:940261. doi: 10.3389/fphar.2022.940261 (PMC9524243; doi:10.3389/fphar.2022.940261)
Supplement: Supplementary file 1 [file Image1.pdf]

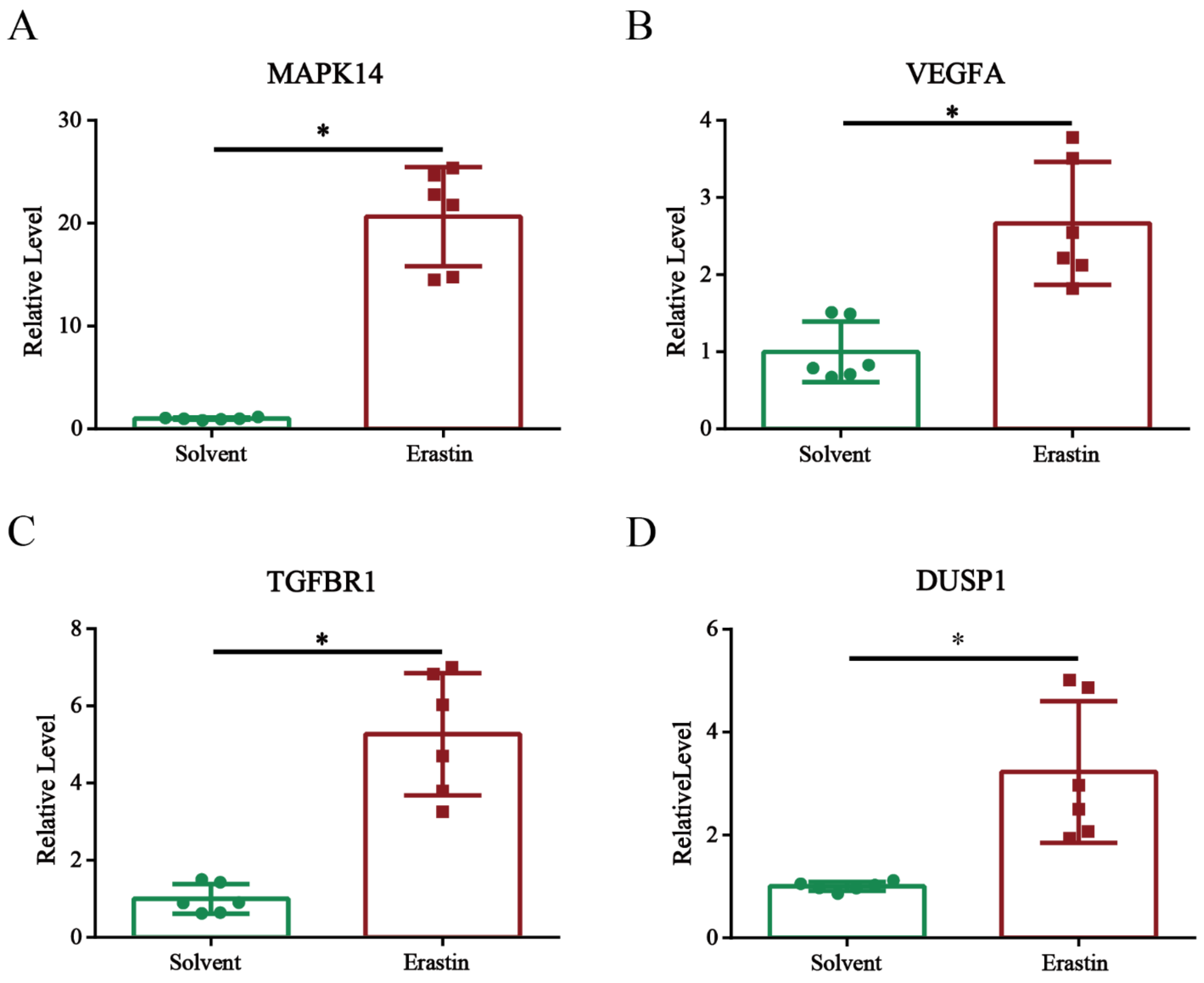

Supplementary Figure 1. qRT-PCR shows that the expression of (A) MAPK14, (B) VEGFA, (C) DUSP1, and (D) TGFBR1 was higher in the Erastin group than in the Solvent group.
